# *Saccharomyces* yeast postbiotics supplemented in feeds for sows and growing pigs for its impact on growth performance of offspring and growing pigs in commercial farm environments

**DOI:** 10.5713/ab.23.0467

**Published:** 2024-02-23

**Authors:** Sung Woo Kim, Marcos Elias Duarte

**Affiliations:** 1 Department of Animal Science, North Carolina State University, Raleigh, NC 27695, USA

**Keywords:** Body Condition Score, Fecal Score, Growth Performance, Pigs, *Saccharomyces* Yeast Postbiotics, Sow

## Abstract

**Objective:**

Three experiments were conducted to evaluate the effects of *Saccharomyces* yeast postbiotics (SYP) in feeds for sows on the growth of offspring (Exp. 1), for nursery pigs on their growth (Exp. 2), and for nursery and finishing pigs on their growth (Exp. 3).

**Methods:**

Exp. 1 had 80 sows at breeding assigned to 4 groups with SYP at 0, 0.050, 0.175, and 0.500 g/kg. Offspring were fed a common diet for 126 d. Exp. 2 had 144 barrows at 8 kg body weight (BW) allotted to CON (no SYP); YPC (SYP at 0.175 g/kg; d 0 to 42); and YPD (SYP at 1.25, 0.75, and 0 g/kg; d 0 to 7, d 8 to 21, and d 22 to 42, respectively) with 8 pens/treatment (6 pigs/pen). Exp. 3 had 96 barrows at 8 kg BW allotted to CON (no SYP); YPN (SYP at 0.175 g/kg; d 0 to 42); YPF (SYP at 0.100 g/kg; d 43 to 119); and YPA (SYP at 0.175 and 0.100 g/kg; d 0 to 42 and d 43 to 119, respectively) with 8 pens/treatment (3 pigs/pen).

**Results:**

In Exp. 1, increasing SYP increased (p<0.05, quadratic) the sow body score (maximum at 0.30% SYP), reduced (p<0.05, quadratic) the days-wean-to-estrus (minimum at 0.27% SYP), and increased (p<0.05) offspring BW at weaning and their average daily gain (ADG) and feed efficiency (G:F) at d 126. In Exp. 2, ADG, average daily feed intake (ADFI), and G:F of YPC were the greatest (p<0.05). The ADG and ADFI of YPD were greater (p<0.05) than CON. Fecal score of YPC and YPD was smaller (p<0.05) than CON. In Exp. 3, YPA had the greatest (p<0.05) ADG and YPN and YPF had greater (p<0.05) ADG than CON.

**Conclusion:**

SYP enhanced sow performance, offspring growth, growth of nursery and growing pigs with the greater efficacy at 0.27 to 0.32 g/kg feed.

## INTRODUCTION

Maternal and perinatal nutrition is critically important for the lifetime growth efficiency of pigs [1,2]. Providing adequate nutrients during mid to late gestation is particularly important because it is the critical time for fetal growth [3,4], fetal muscle fiber proliferation [5,6], and mammary gland development [3,7]. Nutritional status of sows during lactation influences milk production [8,9] and mammary gland growth [10,11] that are the key determinants for the growth and health of new born piglets [12]. The development of musculoskeletal and digestive systems early in a pig’s life is pertinent for efficient growth [4,13,14].

Yeast supplements have long been used in animal feeds as feed additives that can be largely dried yeast including live yeast cells as probiotics, hydrolyzed yeast including inactivated yeast cells often with metabolites as postbiotics, or yeast cell wall components including mannose oligosaccharides and β-glucans as prebiotics possessing specific properties enhancing feed intake [15,16], general health [17–20], growth [21,22], and reproductive performance [8,23,24].

It is interesting to find a possibility of *Saccharomyces* yeast cell content with metabolites possessing signaling components activating target of rapamycin kinases which could be related to coordinating the balance of protein turnover and cell proliferation of mammalian cells including skeletal myocyte hypertrophy [25–28] and intestinal stem cell proliferation [29,30]. *Saccharomyces* yeast cell content with metabolites can be categorized to postbiotics including yeast culture and hydrolyzed yeast.

It is hypothesized that dietary supplementation of *Saccharomyces* yeast postbiotics in feeds for sows and offspring would improve production efficiency of pigs. To test the hypothesis, three experiments were carried out in the commercial pig farm environment to evaluate the effects of supplementing yeast postbiotics in feeds i) for gestating and lactating sows on the growth efficiency of offspring until market, ii) for nursery pigs on their growth efficiency, and iii) for nursery and finishing pigs on their growth efficiency until market.

## MATERIALS AND METHODS

The experiments were carried out at a commercial farm (Wilson, NC, USA). All experimental procedures comply with the Guide for the Care and Use of Agricultural Animals in Research and Teaching [31] and were approved by the Institutional Animal Care and Use Committee (IACUC) of AHPharma Inc. (Hebron, MD, USA) for the safe and humane treatment of animals. *Saccharomyces* yeast probiotics are inactivated *Saccharomyces cerevisiae* with associated metabolites. Initially, *Saccharomyces* yeast was grown in the glycerol based medium for 96 hours to achieve an OD of 100 (optical density measured at 560 nm) and heat treated to dry and inactivate yeast. This *Saccharomyces* yeast postbiotics are commercially available as celluTEIN (Puretein Bioscience LLC, Minneapolis, MN, USA).

### Animals and experimental design

#### Exp. 1

Eighty sows (Smithfield Premium Genetics, 3.7±0.2 parity) at 7 days prior to expected estrus were assigned to 4 groups based on a completely randomized design. Four groups (20 sows/group) were assigned to different supplemental levels of *Saccharomyces* yeast postbiotics (0, 0.050, 0.175, and 0.500 g/kg feed) replacing the same amount of corn. *Saccharomyces* yeast postbiotics were supplemented to the basal diets for gestation and lactation (Table 1) meeting nutrient requirements suggested from NRC [32].

During gestation, sows were given an access to 2 kg assigned gestation diets per day. On 105 d of gestation, sows were moved to farrowing crates and fed the assigned lactation diets (2 kg/d) until farrowing. After farrowing, sows were given an *ad libitum* access of the assigned lactation diets (Table 1).

Upon farrowing, piglets were cross-fostered within a treatment group. Cross-fostering was done only when litter size was greater than 14 or the number of functional mammary glands within 48 h of birth prior to the initiation of regression of non-suckled mammary glands [33–35]. Litters were not given any creep feeds and weaned at 26±1 d of age. Upon weaning, pigs were moved to nursery pens. Two litters within a treatment were placed in a pen. Pigs were fed a common early weaner diet for 7 days until they were 8 kg body weight (BW).

Body condition score of sows were recorded based on the 1 to 5 score scale [36]: 1: emaciated; 2: thin; 3: ideal; 4: fat; and 5: overfat) at breeding, farrowing, and at estrus (subsequent breeding). The number of days from wean to estrus was recorded for each sow.

After 7 days of the early weaner phase, 48 pigs were selected from each treatment group. Four to five pigs with median BW from each pen regardless of sex were selected and housed in new pens. There were 6 pigs per pen using 8 pens per treatment group. All pigs were fed common diets based on a 3 phase feeding program following the Standard Operation Protocol of AHPharma Inc. during nursery and finisher phases (Table 1). Pigs were provided with feed and water *ad libitum*.

The BW of individual pigs were measured at the allotment (7 days after the weaning, d 0 of the study), at the end of nursery phase (d 42 of the study), and at the end of finishing phase (d 126 of the study). Weight gain of pigs were calculated based on the BW of each phase per pen. Feed intake were calculated by measuring the amount of feed consumed during nursery (d 0 to 42 of the study) and finisher (d 42 to 126 of the study) phases. Feed efficiency (G:F) was calculated by dividing the weight gain by the feed intake during nursery and finisher phases. Fecal scores of each pen were recorded using a 1 to 5 scale (1: very firm stool; 2: normal firm stool; 3: moderately loose stool; 4: loose, watery stool; and 5: very watery stool) by visual observation of fresh feces at day 0, 21, and 42 of the study [37,38].

#### Exp. 2

One hundred forty four barrows, weaned at 26±1 d of age, were fed a common diet until they were 8 kg BW and allotted to 3 dietary treatment groups based on a completely randomized design. Therefore, there were 48 barrows housed in 8 pens (6 pigs per pen) per treatment group. Pigs were fed the assigned nursery diet for 42 days (Table 2). Dietary treatments were: i) CON: diets without *Saccharomyces* yeast postbiotics; ii) YPC: CON + yeast postbiotics at 0.175 g/kg from d 0 to 42 (a constant level of supplementation); and iii) YPD: CON + *Saccharomyces* yeast postbiotics at 1.25, 0.75, and 0 g/kg from d 0 to 7, d 8 to 21, and d 22 to 42, respectively (a gradual decrease of supplementation levels). The gradual decrease of supplementation levels in YPD was to achieve similar daily intake of *Saccharomyces* yeast postbiotics whereas pigs in YPC would have increased daily intake of *Saccharomyces* yeast postbiotics as their voluntary feed intake increases as they grow. Pigs were provided with feed and water *ad libitum*. Body weight and feed consumption were measured at d 0, 7, 21, and 42 of the study. Fecal scores of each pen were recorded using a 1 to 5 scale (1: very firm stool; 2: normal firm stool; 3: moderately loose stool; 4: loose, watery stool; and 5: very watery stool) by visual observation of fresh feces at day 7, 21, and 42 of the study [38,39].

#### Exp. 3

Ninety six barrows, weaned at 26±1 d of age, were fed a common diet until they were 8 kg BW and allotted to 4 dietary treatment groups based on a completed randomized design. Therefore, there were 24 barrows housed in 8 pens (3 pigs per pen) per treatment group. Pigs were fed the assigned diet for 119 days based on a 3 phase feeding program (Table 3). Dietary treatments were: i) CON: diets without *Saccharomyces* yeast postbiotics; ii) YPN: CON + *Saccharomyces* yeast postbiotics at 0.175 g/kg from d 0 to 42 of the study; iii) YPF: CON + *Saccharomyces* yeast postbiotics at 0.100 g/kg from d 43 to 119 of the study; and iv) YPA: CON + *Saccharomyces* yeast postbiotics at 0.175 and 0.100 g/kg from d 0 to 42 and d 43 to 119 of the study. Pigs were provided with feed and water *ad libitum*. Body weight and feed consumption were measured at d 0, 42, and 119 of the study. Fecal scores of each pen were recorded using a 1 to 5 scale (1: very firm stool; 2: normal firm stool; 3: moderately loose stool; 4: loose, watery stool; and 5: very watery stool) by visual observation of fresh feces at day 21, and 42 of the study [38,39].

### Statistical analysis

Data were analyzed using the MIXED Procedure of SAS 9.4 (SAS, Cary, NC, USA). In Exp. 1, the levels of *Saccharomyces* yeast postbiotics were considered the fixed effects. Polynomial orthogonal contrast were pre-planned to evaluated the linear and quadratic effects of increasing supplemental levels of *Saccharomyces* yeast postbiotics in sow diets on the growth performance of offspring until marketing. Considering the levels were not equally spaced, the IML Procedure of SAS 9.4 was used to generate the coefficients that was used in the model. In Exp. 2, data were analyzed based on a completely randomized design. Three different feeding programs were the main effect. When a significant difference was found by the main effect, the means were separated using the PDIFF option of the LSMEANS statement. In Exp. 3, data were analyzed based on a completely randomized design. Four different feeding programs were considered as the main effects. When a significant difference was found, the means were separated using the PDIFF option of the LSMEANS statement. The data related with fecal score and diarrhea incidence were analyzed using the npar1way Procedure of SAS 9.4 using Kruskal–Wallis test with Dwass, Steel, Critchlow-Fligner (DSCF) option for pairwise two-sided multiple comparisons [37,40]. Statistical significance and tendency were determined at p<0.05 and 0.05≤p<0.10, respectively.

## RESULTS

### Exp. 1

**Figure 1:**
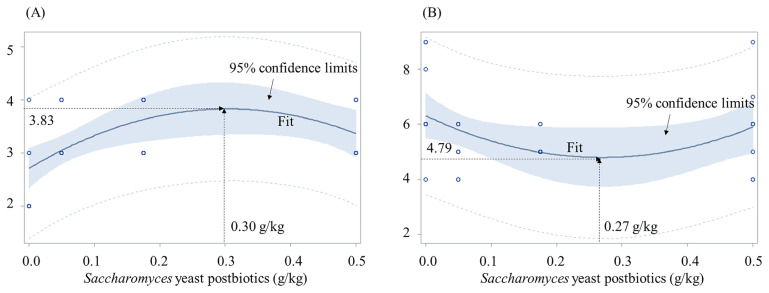
Body condition scores and wean to estrus period of sows fed diets with increasing levels (g/kg) of *Saccharomyces* yeast postbiotics (SYP) during gestation and lactation. (A) Body condition score = 2.71+7.38×SYP–12.15×SYP×SYP (p<0.05 for the intercept, the slope, and the overall model). The maximum body condition score was 3.83 at 0.30 g/kg SYP. Body condition scores were based on the 1 to 5 score scale (Patience and Thacker [36]; 1, emaciated; 2, thin; 3, ideal; 4, fat; and 5, overfat). There were 8 observations for each level and blue dots represent observation (when multiple observations were overlapped, it shows one dot). (B) Wean to estrus period = 6.31–11.27×SYP+20.88×SYP×SYP (p<0.05 for the intercept, the slope, and the overall model). The minimum wean to estrus period was 4.79 at 0.27 g/kg SYP. There were 8 observations for each level and blue dots represent observation (when multiple observations were overlapped, it shows one dot).

At the beginning of the study, the body score of sows at breeding was not different among groups (Table 4). At farrowing, increasing *Saccharomyces* yeast postbiotic supplementation increased (p<0.05, quadratic) the body score of sows (maximum of 3.83 at 0.30% *Saccharomyces* yeast postbiotics, Figure 1A). Increasing *Saccharomyces* yeast postbiotic supplementation reduced (p<0.05, quadratic) the number of days from wean to estrus reduced (minimum of 4.79 days at 0.27% *Saccharomyces* yeast postbiotics, Figure 1B).

**Figure 2:**
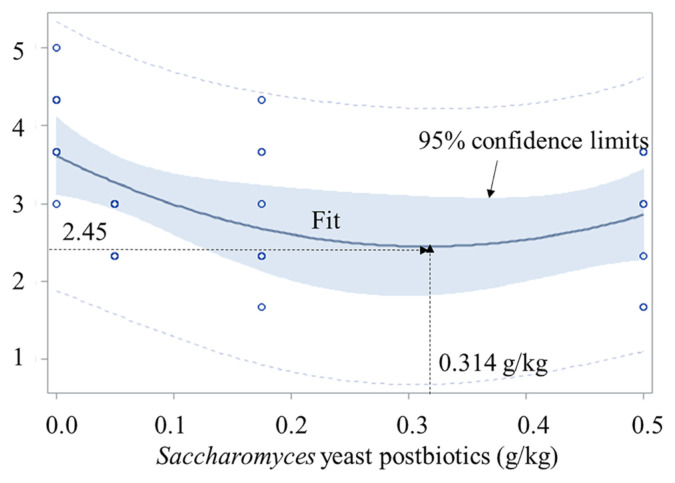
Fecal scores of offspring at d 47 of age from sows fed diets with increasing levels (g/kg) of *Saccharomyces* yeast postbiotics (SYP) during gestation and lactation. Fecal score = 3.61–7.40×SYP+ 11.79×SYP×SYP (p<0.05 for the intercept, the slope, and the overall model). The minimum fecal score was 2.45 at 0.314 g/kg SYP. Fecal scores were based on the 1 to 5 scale (1, very firm stool; 2, normal firm stool; 3, moderately loose stool; 4, loose, watery stool; and 5, very watery stool) by visual observation of fresh feces [38,39]. There were 8 observations for each level and blue dots represent observation (when multiple observations were overlapped, it shows one dot).

Increasing *Saccharomyces* yeast postbiotic supplementation to sows increased (p<0.05) the BW of offspring at the time of allotment (Table 5). Increasing *Saccharomyces* yeast postbiotic supplementation to sows increased (p<0.05) average daily gain (ADG) of offspring during nursery period (d 0 to 42 of the study), finisher period (d 42 to 126 of the study), and the entire 126 days. Increasing *Saccharomyces* yeast postbiotic supplementation to sows increased (p<0.05) average daily feed intake (ADFI) of offspring during nursery period (d 0 to 42 of the study), and tended to increase (p = 0.057) ADFI of offspring during the entire 126 days. Increasing *Saccharomyces* yeast postbiotic supplementation to sows increased (p<0.05) G:F of offspring during nursery period (d 0 to 42 of the study), finisher period (d 42 to 126 of the study), and the entire 126 days. Increasing *Saccharomyces* yeast postbiotic supplementation to sows tended to reduce (p = 0.057) fecal score of offspring at d 0 and reduced (p<0.05) fecal score of offspring at 21 and 42 of the study (Table 6). On d 42 of the study, fecal score was the lowest (2.45, p<0.05) at 0.314% *Saccharomyces* yeast postbiotics (Figure 2).

### Exp. 2

The BW of pigs at the time of allotment did not differ among treatment groups (Table 7). During d 0 to 7, ADG and ADFI of pigs in YPD were the greatest (p<0.05). The ADG and ADFI of pigs in YPC were greater (p<0.05) than pigs in CON. During d 7 to 21, ADG and ADFI of pigs in YPC and YPD were greater (p<0.05) than pigs in CON. During d 21 to 42, ADG and ADFI of pigs in YPC were the greatest (p<0.05). The ADG and ADFI of pigs in YPD were greater (p<0.05) than pigs in CON. During the entire 42 d period, ADG and ADFI of pigs in YPC were the greatest (p<0.05), and the ADG and ADFI of pigs in YPD were greater (p<0.05) than pigs in CON.

During d 0 to 7, G:F of pigs in YPD were the greatest (p< 0.05) and G:F of pigs in YPC were greater (p<0.05) than pigs in CON. During d 7 to 21, d 21 to 42, and the entire period, G:F of pigs in YPC and YPD were greater (p<0.05) than pigs in CON. During the entire 42 d period, G:F of pigs in YPC were the greatest (p<0.05). Fecal score of pigs in YPC and YPD was smaller (p<0.05) than pigs in CON on d 7, 21, and 42 of the study (Table 8).

### Exp. 3

The BW of pigs at the time of allotment did not differ among treatment groups. During d 0 to 42 (nursery period), ADG of pigs in YPN and YPA was greater (p<0.05) than pigs in CON and YPF (Table 9). During d 42 to 119 (finisher period), ADG of pigs in YPA was the greatest (p<0.05) whereas that in CON was the lowest (p<0.05). The ADG of pigs in YPF was greater (p<0.05) than pigs in YPN. During the entire 119 d period, ADG of pigs in YPA was the greatest (p<0.05) and ADG of pigs in YPN and YPF was greater (p<0.05) then pigs in CON. The ADFI of pigs was not different among treatment groups.

During d 0 to 42 (nursery period), G:F of pigs in YPN and YPA was greater (p<0.05) than pigs in CON and YPF. During d 42 to 119 (finisher period) and the entire 119 d period, G:F of pigs in YPA was the greatest (p<0.05) whereas that in CON was the lowest (p<0.05). The G:F of pigs in YPF was greater (p<0.05) than pigs in YPN. Fecal score of pigs in YPN and YPA was lower (p<0.05) than pigs in CON and YPF at d 21 and 42 of the study. There was no difference in fecal score between pigs in CON and YPF on d 21 and 42 of the study (Table 10)

## DISCUSSION

This study evaluated the effects of *Saccharomyces* yeast postbiotics, an inactivated yeast based feed additive to diets for sows and growing pigs on production performance of sows, their offspring, and growing pigs. The yeast feed additive used in this study is derived from the fermentation products of *Saccharomyces cerevisiae* together with inactivated yeast cells. This kind of additives belongs to the ‘yeast’ category in the section 96 of the AAFCO definition [41] and this category includes primary dried yeast (section 96.1), grain distillers dried yeast (section 96.5), hydrolyzed yeast (section 96.12), active dry yeast (section 96.2), yeast culture (section 96.8), etc. This yeast term by AAFCO is rather broad including prebiotics, probiotics, and postbiotics derived from yeast.

The International Scientific Association for Probiotic and Prebiotics (ISAPP) defined postbiotics as “preparations of inanimate microorganisms and/or their components that confers a health benefit on the host”. Postbiotics belonging to the section 96 of the AAFCO definition are yeast culture (96.8) and hydrolyzed yeast (96.12) whereas other yeast categories may include active yeast cells belonging to probiotics. Postbiotics include heat-treated, tyndallized (heat-killed), or inanimate microorganisms.

Inactivated yeast and its culture have long been used in animal feeding as postbiotic supplements. Supplementation of yeast culture in feeds for breeding animals enhanced voluntary feed intake benefiting maternal body condition, fetal growth and milk yield in both dairy cattle [42–44] and sows [8,23,24]. This study supported these outcomes from the previous works. The pigs from sows fed diets with *Saccharomyces* yeast postbiotics had increased BW at weaning and the sows maintained enhanced body condition scores and reduced wean to estrus period when *Saccharomyces* yeast postbiotics were supplemented at 0.27 to 0.30 g/kg feed during gestation and lactation (Figure 1; Table 4). This study showed improvement in sow body condition score from 2.7 to 3.8 when the supplementation of *Saccharomyces* yeast postbiotics was increased from 0 to 0.30 g/kg. The number of days from wean to estrus was reduced from 3.6 to 2.5 d when the supplementation of *Saccharomyces* yeast postbiotics was increased from 0 to 0.27 g/kg. Considering that the sow body condition score of 2 is considered ‘thin sow’ and 3 is ‘ideal sow’ [36], increase of the score from 2.7 to 3.8 is beneficial to pig production as it was also resulting in reduced number of days from wean to estrus by 0.9 d. An article from National Hog Farmer [45] in the US reported the cost of non-productive sow day to be in a range of $2.0 and $3.6 when estimated based on febed cost and production cost in the US. The reduction by 0.9 d from this study is significant benefit to pig producers depending on the size of the production and the cost of feedstuffs.

Fecal score of offspring at d 42 of age was reduced from 3.6 to 2.5 (Figure 2; Table 6). The fecal score of 2 is dedicated to ‘normal firm stool’ whereas 4 is ‘loose [38,39]. The reduction of the fecal score from 3.6 to 2.5 is considered enhanced intestinal health and often related to increase in growth performance. Similar reduction in fecal score enhanced intestinal health by reducing tumour necrosis factor-α by 30%, protein carbonyl by 39%, and immunoglobulin G (IgG) by 38% resulting in increase of ADG by 26% [46]; enhanced intestinal health by reducing malondialdehyde by 69% and interleukin-6 by 30% resulting in increase of ADG by 20% [38]; and enhanced intestinal health by reducing protein carbonyl by 32%, interleukin-8 by 45%, and IgA by 29% resulting in increase of ADG by 31% [47]. Body weight of offspring from sows fed diets with *Saccharomyces* yeast postbiotics was increased by 2.0% at 7 d after weaning which was maintained at market weight of 130 kg with an increase of 3.5% (Table 5). It has been well demonstrated that the increase in BW at weaning would cause increase in market weight or reduce the days to reach the market weight [1,48]. In this study, supplementation of *Saccharomyces* yeast postbiotics to sow feeds at 0.27 to 0.31 g/kg showed beneficial effects by improving reproductive performance of sows and growth performance of offspring from sows fed diets with *Saccharomyces* yeast postbiotics. Kim et al [23], Shen et al [8,49], and Zhao et al [50] support the outcomes from this study.

Yeast postbiotics or yeast culture have been used in nursery diets enhancing growth possible by increasing feed intake and improving intestinal health from weaning stress [15,16, 22]. In this study, feeding diets with yeast postbiotics for 42 day increased ADFI of nursery pigs by 13% that is similar to previous findings (7% to 25%) [15,22]. The enhanced feed intake could be a reflection of healthy intestine as indicated by reduced fecal score by 24%. Previous studies used yeast cell wall components in nursery diets demonstrating their prebiotic effects, antibacterial properties, and toxin binding property [18–20,51]. These BW gain was further increased by dietary supplementation of yeast postbiotics in this study (22%) that is similar or a bit higher than previous findings (12% to 21%) [15,22] resulting in enhanced feed efficiency (7%). Yeast cell wall components have also been used in nursery diets for their prebiotic effects, antibacterial properties, and toxin binding property [18–20,51]. A thorough review of bioactive compounds in microbial cell wall [52] characterized the composition of typical yeast cell wall including mannoprotein (35% to 40%), 1,3-β-glucan (50% to 55%), 1,6-β-glucan (5% to 10%), chitin (up to 3%) with immunomodulatory properties, antibacterial properties potentially beneficial to maintain healthy intestine and to reduce the occurrence of diarrhea as observed in this study. Yeast cell contents, however, are shown to be a good source of protein and also rich in nucleotides improving intestinal health when fed to nursery pigs reducing pathogenic invasion [21,53,54].

In conclusion, this study demonstrated that dietary supplementation of *Saccharomyces* yeast postbiotics to diets for gestating sows, lactating sows, nursery pigs, and growing-finishing pigs enhanced reproductive performance, growth of progeny, growth of nursery and growing pigs. Supplementation of *Saccharomyces* yeast postbiotics at 0.27 to 0.32 g/kg feed was the most effectively for these production traits.

## Figures and Tables

**Table 1:** Composition of experimental diets used in Exp. 1

Feedstuff (%)	Sow		Nursery	Finisher 1	Finisher 2
	
	Gestation	Lactation	d 0 to 42	d 42 to 56	d 56 to 119
Yellow corn	74.70	66.51	64.57	72.20	78.08
Soybean meal, 47% CP	17.44	25.77	28.60	23.60	18.50
L-Lys HCl	0.24	0.35	0.51	0.34	0.29
DL-Met	-	-	0.17	0.10	0.05
L-Thr	-	0.11	0.17	0.10	0.05
A-V blend oil	3.96	3.60	2.20	0.60	0.05
Limestone	1.52	1.52	1.78	1.33	0.98
Dicalcium phosphate	1.51	1.51	1.37	1.17	1.07
Salt	0.45	0.45	0.45	0.40	0.35
Vitamin and mineral premix^1)^	0.13	0.13	0.13	0.13	0.13
Supplement^2)^	0.05	0.05	-	-	-
Total	100.00	100.00	100.00	100.00	100.00
Calculated composition					
Dry matter (%)	89.5	89.6	89.4	89.2	89.0
ME (kcal/kg)	3,455	3,430	3,359	3,300	3,316
Crude protein (%)	14.7	18.2	19.7	17.7	15.6
SID Lys (%)	0.79	1.08	1.27	1.02	0.86
SID Met + Cys (%)	0.43	0.51	0.70	0.60	0.51
SID Thr (%)	0.44	0.66	0.76	0.63	0.51
SID Trp (%)	0.14	0.19	0.20	0.18	0.15
Calcium (%)	0.87	0.89	0.97	0.76	0.60
STTD P (%)	0.41	0.43	0.41	0.36	0.33
Total P (%)	0.64	0.67	0.66	0.60	0.56

ME, metabolizable energy; SID, standardized ileal digestibility; STTD, standardized total tract digestibility.

1) The vitamin-mineral premix provided the following per kilogram of complete diet: 3.96 mg of Mn as manganous oxide; 16.5 mg of Fe as ferrous sulfate; 16.5 mg of Zn as zinc sulfate; 1.65 mg of Cu as copper sulfate; 0.30 mg of I as ethylenediamine dihydroiodide; 0.30 mg of Se as sodium selenite; 8,228 IU of vitamin A as vitamin A acetate; 1,173 IU of vitamin D_3_; 47 IU of vitamin E; 0.03 mg of vitamin B_12_; 5.88 mg of riboflavin; 23.52 mg of D-pantothenic acid as calcium panthonate; 35.27 mg of niacin; 0.24 mg of biotin; 1.76 mg folic acid; 3.88 mg menadione.

2) Supplement (0.05%) is composed of *Saccharomyces* yeast postbiotics (celluTEIN, Puretein Bioscience LLC, Minneapolis, MN, USA) and corn: by increasing *Saccharomyces* yeast postbiotics from 0% 0.0050%, 0.0175%, and 0.0500% and reducing corn from 0.0500%, 0.0450%, 0.0325%, and 0%, respectively.

**Table 2:** Composition of experimental diets used in Exp. 2

Feedstuff (%)	Nursery, 0 to 42 d
Yellow corn	62.87
Soybean meal, 47% CP	29.00
L-Lys HCl	0.51
DL-Met	0.17
L-Thr	0.17
A-V blend oil	3.30
Limestone	1.78
Dicalcium phosphate	1.37
Salt	0.45
Vitamin and mineral premix^1)^	0.13
Supplement^2)^	0.25
Total	100.00
Calculated composition	
Dry matter (%)	89.7
ME (kcal/kg)	3,406
Crude protein (%)	19.8
SID Lys (%)	1.28
SID Met + Cys (%)	0.70
SID Thr (%)	0.76
SID Trp (%)	0.20
Calcium (%)	0.97
STTD P (%)	0.41
Total P (%)	0.66

ME, metabolizable energy; SID, standardized ileal digestibility; STTD, standardized total tract digestibility.

1) The vitamin-mineral premix provided the following per kilogram of complete diet: 3.96 mg of Mn as manganous oxide; 16.5 mg of Fe as ferrous sulfate; 16.5 mg of Zn as zinc sulfate; 1.65 mg of Cu as copper sulfate; 0.30 mg of I as ethylenediamine dihydroiodide; 0.30 mg of Se as sodium selenite; 8,228 IU of vitamin A as vitamin A acetate; 1,173 IU of vitamin D_3_; 47 IU of vitamin E; 0.03 mg of vitamin B_12_; 5.88 mg of riboflavin; 23.52 mg of D-pantothenic acid as calcium panthonate; 35.27 mg of niacin; 0.24 mg of biotin; 1.76 mg folic acid; 3.88 mg menadione.

2) Supplement (0.05%) is composed of *Saccharomyces* yeast postbiotics (celluTEIN, Puretein Bioscience LLC, Minneapolis, MN, USA) and corn with varying composition depending on dietary treatments: i) CON: 0.25% corn; ii) YPC: 0.0175% *Saccharomyces* yeast postbiotics and 0.2325% corn from d 0 to 42 (a constant level of supplementation); iii) YPD: *Saccharomyces* yeast postbiotics and corn at 0.125% and 0.125% from d 0 to 7, 0.075% and 0.175% from d 7 to 21, and 0% and 0.25% from d 21 to 42, respectively.

**Table 3:** Composition of experimental diets used in Exp. 3

Feedstuff (%)	Nursery	Finisher 1	Finisher 2

	0 to 42 d	42 to 56 d	56 to 119 d
Yellow corn	64.57	72.20	78.08
Soybean meal, 47% CP	28.60	23.60	18.50
L-Lys HCl	0.51	0.34	0.29
DL-Met	0.17	0.10	0.05
L-Thr	0.17	0.10	0.05
A-V blend oil	2.20	0.60	0.05
Limestone	1.78	1.33	0.98
Dicalcium phosphate	1.37	1.17	1.07
Salt	0.45	0.40	0.35
Vitamin and mineral premix^1)^	0.13	0.13	0.13
Supplement^2)^	0.05	0.05	0.05
Total	100.00	100.00	100.00
Calculated composition			
Dry matter (%)	89.4	89.2	89.0
ME (kcal/kg)	3,359	3,300	3,316
Crude protein (%)	19.7	17.7	15.6
SID Lys (%)	1.27	1.02	0.86
SID Met + Cys (%)	0.70	0.60	0.51
SID Thr (%)	0.76	0.63	0.51
SID Trp (%)	0.20	0.18	0.15
Calcium (%)	0.97	0.76	0.60
STTD P (%)	0.41	0.36	0.33
Total P (%)	0.66	0.60	0.56

CP, crude protein; ME, metabolizable energy; SID, standardized ileal digestibility; STTD, standardized total tract digestibility.

1) The vitamin-mineral premix provided the following per kilogram of complete diet: 3.96 mg of Mn as manganous oxide; 16.5 mg of Fe as ferrous sulfate; 16.5 mg of Zn as zinc sulfate; 1.65 mg of Cu as copper sulfate; 0.30 mg of I as ethylenediamine dihydroiodide; 0.30 mg of Se as sodium selenite; 8,228 IU of vitamin A as vitamin A acetate; 1,173 IU of vitamin D_3_; 47 IU of vitamin E; 0.03 mg of vitamin B_12_; 5.88 mg of riboflavin; 23.52 mg of D-pantothenic acid as calcium panthonate; 35.27 mg of niacin; 0.24 mg of biotin; 1.76 mg folic acid; 3.88 mg menadione.

2) Supplement (0.05%) is composed of *Saccharomyces* yeast postbiotics (celluTEIN, Puretein Bioscience LLC, Minneapolis, MN, USA) and corn with varying composition depending on dietary treatments: i) CON: 0.25% corn; ii) YPN: 0.0175% *Saccharomyces* yeast postbiotics and 0.0325% corn from d 0 to 42 of the study; iii) YPF: 0.01% *Saccharomyces* yeast postbiotics and 0.04% corn from d 43 to 119 of the study; iv) YPA: 0.0175% *Saccharomyces* yeast postbiotics and 0.0325% corn from d 0 to 42 of the study and 0.01% *Saccharomyces* yeast postbiotics and 0.04% corn from d 43 to 119 of the study.

**Table 4:** Body condition scores^1)^ and wean to estrus period of sows fed diets with increasing levels of *Saccharomyces* yeast postbiotics during gestation and lactation

Item	*Saccharomyces* yeast postbiotics^2)^ (g/kg)				SEM	p-value	
	
	0	0.05	0.175	0.500		Linear	Quadratic
Initial	3.38	3.63	3.63	3.63	0.18	0.530	0.483
Farrowing	2.50	3.30	3.50	3.38	0.21	0.091	0.038
Estrus	3.63	3.75	3.75	3.63	0.17	0.801	0.568
Wean to estrus (d)	6.75	5.13	5.25	5.88	0.46	0.689	0.028

1) Body condition scores: based on the 1 to 5 score scale (Patience and Thacker [36]; 1, emaciated; 2, thin; 3, ideal; 4, fat; 5, overfat).

2) *Saccharomyces* yeast postbiotics (celluTEIN, Puretein Bioscience LLC, Minneapolis, MN, USA) was supplemented to sows during gestation and lactation.

**Table 5:** Growth performance of nursery pigs from sows fed diets with increasing levels of *Saccharomyces* yeast postbiotics during gestation and lactation

Item	*Saccharomyces* yeast postbiotics^1)^ (g/kg)				SEM	p-value	
	
	0	0.05	0.175	0.500		Linear	Quadratic
Body weight (kg)							
d 0	8.09	8.10	8.20	8.24	0.02	<0.001	0.037
d 7	11.16	11.29	11.39	11.48	0.09	0.028	0.289
d 21	22.14	22.45	22.82	23.09	0.07	<0.001	0.001
d 42	42.76	43.29	43.83	44.11	0.18	<0.001	0.011
d 126	127.54	129.76	130.89	132.05	0.58	<0.001	0.022
Average daily gain (kg/d)							
d 0 to 7	0.439	0.456	0.456	0.463	0.012	0.256	0.580
d 7 to 21	0.784	0.797	0.816	0.830	0.008	<0.001	0.100
d 21 to 42	0.982	0.992	1.001	1.001	0.009	0.193	0.273
d 0 to 42	0.826	0.838	0.848	0.854	0.004	<0.001	0.024
d 42 to 126	1.009	1.037	1.029	1.047	0.006	0.002	0.372
d 0 to 126	0.948	0.967	0.974	0.983	0.005	<0.001	0.026
Average daily feed intake (kg/d)							
d 0 to 7	0.756	0.776	0.773	0.778	0.019	0.575	0.690
d 7 to 21	1.473	1.459	1.479	1.489	0.022	0.279	0.985
d 21 to 42	3.129	3.072	3.027	2.992	0.034	0.012	0.213
d 0 to 42	2.181	2.152	2.135	2.122	0.016	0.029	0.225
d 42 to 126	2.836	2.831	2.810	2.796	0.023	0.192	0.665
d 0 to 126	2.618	2.604	2.585	2.571	0.017	0.057	0.427
Feed efficiency (G:F)							
d 0 to 7	0.581	0.588	0.589	0.595	0.002	<0.001	0.274
d 7 to 21	0.533	0.546	0.552	0.557	0.003	<0.001	0.013
d 21 to 42	0.314	0.323	0.331	0.335	0.002	<0.001	0.001
d 0 to 42	0.379	0.390	0.397	0.403	0.002	<0.001	<0.001
d 42 to 126	0.358	0.366	0.370	0.375	0.002	<0.001	0.019
d 0 to 126	0.362	0.371	0.377	0.382	0.001	<0.001	<0.001

SEM, standard error of the mean.

1) *Saccharomyces* yeast postbiotics (celluTEIN, Puretein Bioscience LLC, Minneapolis, MN, USA) was supplemented to sows during gestation and lactation.

**Table 6:** Fecal scores of nursery pigs from sows fed diets with increasing levels of *Saccharomyces* yeast postbiotics during gestation and lactation

Fecal score^1)^	*Saccharomyces* yeast postbiotics^2)^ (g/kg)				SEM	p-value	
	
	0	0.05	0.175	0.500		Linear	Quadratic
d 0	3.9	3.1	3.4	3.2	0.2	0.057	0.156
d 21	3.9	2.7	2.7	3.2	0.2	0.021	0.315
d 42	4.0	2.8	2.9	2.7	0.2	0.033	0.007

SEM, standard error of the mean.

1) Fecal scores of each pen were recorded using a 1 to 5 scale (1: very firm stool; 2: normal firm stool; 3: moderately loose stool; 4: loose, watery stool; 5: very watery stool) by visual observation of fresh feces [38,39].

2) *Saccharomyces* yeast postbiotics (celluTEIN, Puretein Bioscience LLC, Minneapolis, MN, USA) was supplemented to sows during gestation and lactation.

**Table 7:** Growth performance of pigs fed diets with different levels of *Saccharomyces* yeast postbiotics during the 42 d nursery period

Items	Feeding program^1)^			SEM	p-value

	CON	YPC	YPD		
Body weight (kg)					
d 0	8.03	8.04	8.05	0.03	0.899
d 7	9.16^c^	9.56^b^	9.82^a^	0.07	<0.001
d 21	13.54^b^	14.69^a^	14.82^a^	0.08	<0.001
d 42	22.28^c^	25.86^a^	24.92^b^	0.13	<0.001
Average daily gain (kg/d)					
d 0 to 7	0.162^c^	0.218^b^	0.267^a^	0.009	<0.001
d 7 to 21	0.313^b^	0.366^a^	0.351^a^	0.008	<0.001
d 21 to 42	0.416^c^	0.532^a^	0.481^b^	0.006	<0.001
d 0 to 42	0.339^c^	0.424^a^	0.402^b^	0.003	<0.001
Average daily feed intake (kg/d)					
d 0 to 7	0.309^c^	0.409^b^	0.493^a^	0.018	<0.001
d 7 to 21	0.642^b^	0.726^a^	0.697^a^	0.016	0.002
d 21 to 42	1.506^c^	1.753^a^	1.592^b^	0.021	<0.001
d 0 to 42	0.767^c^	0.895^a^	0.845^b^	0.007	<0.001
Feed efficiency (G:F)					
d 0 to 7	0.525^c^	0.533^b^	0.542^a^	0.002	<0.001
d 7 to 21	0.487^b^	0.504^a^	0.503^a^	0.003	0.006
d 21 to 42	0.276^b^	0.302^a^	0.304^a^	0.003	0.004
d 0 to 42	0.442^b^	0.474^a^	0.475^a^	0.002	<0.001

SEM, standard error of the mean.

1) CON: diets without yeast postbiotics; YPC: CON + yeast postbiotics at 0.175 g/kg from d 0 to 42 (a constant level of supplementation); and YPD: CON + yeast postbiotics at 1.25, 0.75, and 0 g/kg from d 0 to 7, d 7 to 21, and d 21 to 42, respectively (a gradual decrease of supplementation levels).

a–c Means lacking common superscripts differ (p<0.05).

**Table 8:** Fecal score of pigs fed diets with different levels of *Saccharomyces* yeast postbiotics during the 42 d nursery period

Fecal score^1)^	Feeding program^2)^			SEM	p-value

	CON	YPC	YPD		
d 7	4.1^a^	3.3^b^	3.5^b^	0.1	<0.001
d 21	4.1^a^	3.2^b^	3.3^b^	0.1	0.007
d 42	4.2^a^	3.3^b^	3.1^b^	0.2	0.002

SEM, standard error of the mean.

1) Fecal scores of each pen were recorded using a 1 to 5 scale (1: very firm stool; 2: normal firm stool; 3: moderately loose stool; 4: loose, watery stool; 5: very watery stool) by visual observation of fresh feces [38,39].

2) CON: diets without yeast postbiotics; YPC: CON + yeast postbiotics at 0.175 g/kg from d 0 to 42 (a constant level of supplementation); YPD: CON + yeast postbiotics at 1.25, 0.75, and 0 g/kg from d 0 to 7, d 8 to 21, and d 22 to 42, respectively (a gradual decrease of supplementation levels).

a,b Means lacking common superscripts differ (p<0.05).

**Table 9:** Growth performance of pigs fed diets with different levels of *Saccharomyces* yeast postbiotics during the 119 d nursery and finisher periods

Items	Feeding program^1)^				SEM	p-value

	CON	YPN	YPF	YPA		
Body weight (kg)						
d 0	7.50	7.53	7.45	7.51	0.05	0.779
d 42	32.78^b^	34.25^a^	32.76^b^	34.17^a^	0.28	<0.001
d 119	115.92^c^	120.26^b^	121.04^b^	126.05^a^	0.72	<0.001
Average daily gain (kg/d)						
d 0 to 42	0.602^b^	0.636^a^	0.603^b^	0.635^a^	0.006	<0.001
d 42 to 119	1.080^d^	1.117^c^	1.146^b^	1.193^a^	0.009	<0.001
d 0 to 119	0.911^c^	0.947^b^	0.955^b^	0.996^a^	0.006	<0.001
Average daily feed intake (kg/d)						
d 0 to 42	1.251	1.283	1.251	1.286	0.016	0.192
d 42 119	3.096	3.143	3.091	3.142	0.028	0.404
d 0 to 119	2.445	2.487	2.442	2.487	0.018	0.156
Feed efficiency (G:F)						
d 0 to 42	0.481^b^	0.496^a^	0.482^b^	0.494^a^	0.002	<0.001
d 42 119	0.349^d^	0.356^c^	0.371^b^	0.380^a^	0.002	<0.001
d 0 to 119	0.373^d^	0.381^c^	0.391^b^	0.401^a^	0.002	<0.001

SEM, standard error of the mean.

1) CON: diets without yeast postbiotics; YPN: CON + yeast postbiotics at 0.175 g/kg from d 0 to 42 of the study; YPF: CON + yeast postbiotics at 0.100 g/kg from d 43 to 119 of the study; YPA: CON + yeast postbiotics at 0.175 and 0.100 g/kg from d 0 to 42 and d 43 to 119 of the study.

a–d Means lacking common superscripts differ (p<0.05).

**Table 10:** Diarrhea incidence of pigs fed diets with different levels of *Saccharomyces y*east postbiotics during the 119 d nursery and finisher periods

Fecal score^1)^	Feeding program^2)^				SEM	p-value

	CON	YPN	YPF	YPA		
d 21	3.8^a^	3.2^b^	4.1^a^	3.1^b^	0.2	0.001
d 42	4.1^a^	3.2^b^	4.2^a^	3.2^b^	0.2	<0.001

SEM, standard error of the mean.

1) Fecal scores of each pen were recorded using a 1 to 5 scale (1: very firm stool; 2: normal firm stool; 3: moderately loose stool; 4: loose, watery stool; 5: very watery stool) by visual observation of fresh feces [38,39].

2) CON: diets without yeast postbiotics; YPN: CON + yeast postbiotics at 0.175 g/kg from d 0 to 42 of the study; YPF: CON + yeast postbiotics at 0.100 g/kg from d 43 to 119 of the study; YPA: CON + yeast postbiotics at 0.175 and 0.100 g/kg from d 0 to 42 and d 43 to 119 of the study

a,b Means lacking common superscripts differ (p<0.05).
